# Global Farm Platform: Pioneer Farm Agroecosystem Research as a long-term hub-and-spoke approach for livestock-integrated systems in the Upper Mississippi River Basin, United States

**DOI:** 10.1093/af/vfag023

**Published:** 2026-07-07

**Authors:** Andrew D Cartmill, Dennis L Busch

**Affiliations:** School of Agriculture and Environment, Massey University, Palmerston North, New Zealand; School of Agriculture, University of Wisconsin-Platteville, Platteville, WI 53818

**Keywords:** crop–livestock integration, dairy systems, environmental performance, soil health, water quality

Implications

- Effective evaluation of agroecosystem sustainability requires research infrastructures capable of capturing long-term interactions among soil, water, biodiversity, and management.
- Hub-and-spoke research architectures provide a scalable framework for integrating controlled experimentation with real-world farming complexity, improving the adoption potential of agroecological innovations.
- Embedding farmer-led watershed groups within research networks enhances the legitimacy, relevance, and scalability of outcomes, particularly for nutrient management, water quality, and ecosystem services.
- Long-term, research platforms such as the Pioneer Farm Agroecosystem Research platform are essential for informing policy, supporting adaptive management, and enabling cross-scale synthesis.

## Introduction

Agroecological research operates at the intersection of biophysical processes, management decisions, and institutional constraints, requiring approaches that move beyond traditional experimental paradigms to address real-world agricultural complexity. In many regions, these agroecosystems are structured around crop–livestock systems, where animals play a central role in mediating the flow of energy and nutrients across landscapes (Lemaire et al., 2014; Martin et al., 2016). As a result, agricultural sustainability is closely linked to livestock production, feed systems, manure management, and their interactions with soil and water processes. In confined dairy and mixed crop–livestock systems, livestock influence nutrient cycling primarily through feed production, manure collection, storage, and land application, linking animal production directly to soil fertility and environmental outcomes (Steinfeld and Wassenaar, 2007; Tully and Ryals, 2017). These linkages are particularly important for understanding water quality and nutrient loss pathways, as the timing, placement, and rate of nutrient applications interact with soil conditions and climatic variability to determine both nutrient use efficiency and environmental risk (Meals et al., 2010; Saha et al., 2023). Nutrient redistribution through manure management introduces spatial and temporal complexity, often creating uneven patterns of nutrient accumulation and loss across fields and landscapes (Méité et al., 2024; Xu et al., 2026).

Understanding agroecosystem sustainability under these conditions requires research infrastructures capable of capturing cumulative changes in soil, water, biodiversity, and ecosystem function within working agricultural landscapes. Short-term or isolated trials, while essential for identifying mechanistic relationships, are often insufficient for detecting system-level dynamics arising from interactions among management, environmental processes, and socio-economic drivers (Pereponova et al., 2023; Jones et al., 2025). This limitation is particularly evident in livestock-integrated systems, where feedbacks among feed production, manure management, soil processes, and hydrological pathways evolve over time and are shaped by operational constraints and decision-making at the farm scale (Cesoniene et al., 2019; Tullo et al., 2019).

In response to these limitations, there is increasing recognition of the need for integrated research platforms that combine long-term measurement, stakeholder engagement, and networked synthesis. Hub-and-spoke research architectures offer a pragmatic alternative, enabling coordinated experimentation while supporting distributed, stakeholder-led learning. At Pioneer Farm (University of Wisconsin-Platteville), a hub-and-spoke framework provides the foundation for integrating long-term agroecological research with farmer practice and landscape-scale outcomes. Aligned with the United States Department of Agriculture (USDA) Long-Term Agroecosystem Research (LTAR) Network and the Global Farm Platform (GFP), the Pioneer Farm Agroecosystem Research (PFAR) platform prioritizes co-production over top-down knowledge transfer, positioning research as a collaborative process that integrates scientific and experiential knowledge. The platform functions as a methodological and relational anchor, supporting long-term experimentation, shared data standards, and institutional continuity.

In this paper, we describe the PFAR platform as a transferable model for long-term, production-representative agroecosystem research that integrates livestock-relevant environmental monitoring, farmer-led watershed engagement, and networked governance across local, national, and international scales. We argue that such platforms are essential for advancing understanding of crop–livestock systems, particularly in relation to nutrient cycling, water quality, and environmental performance.

## The Need for Long-Term Agroecosystem Research

Understanding agroecosystem function requires temporal perspectives that extend well beyond the duration of most conventional research studies. Agricultural systems are inherently dynamic, shaped by interactions among soil processes, hydrology, biodiversity, climate variability, and management decisions that unfold over multiple years to decades (Gordon et al., 2008). While short-term experiments have been instrumental in identifying mechanistic relationships and informing component-level interventions, they are often insufficient for capturing system-level outcomes.

Many of the processes that underpin agroecosystem sustainability operate over extended timeframes. Changes in soil organic matter reflect the balance of inputs and losses accumulated over years or decades and are strongly influenced by climatic variability and management history (da Silva et al., 2025). Similarly, nutrient dynamics, including the accumulation, transformation, and transport of nitrogen and phosphorus, often exhibit lagged responses, particularly in landscapes with strong hydrological connectivity (Wang et al., 2023). Biodiversity responses may also depend on sustained management regimes, with shifts in species composition and functional diversity emerging gradually over time (Dudley and Alexander, 2017). Hydrological processes further complicate these dynamics, as pathways linking fields to surface and groundwater systems can amplify or delay the expression of management effects (Bagagnan et al., 2026). For many soil and water indicators, meaningful assessment requires long-term measurement over at least one to two decades, enabling the detection of trends across climatic variability and changes in management (Bai et al., 2018).

Despite this clear need, current research funding models remain poorly aligned with the temporal requirements of agroecosystem science. Competitive funding structures are typically organized around short project cycles, often spanning 3 to 5 years, with a strong emphasis on novelty, rapid outputs, and discrete deliverables. While this model supports innovation and responsiveness, it provides limited incentives for the establishment and maintenance of long-term research infrastructure. As a result, researchers are frequently constrained to designing studies that fit within funding timelines rather than those that reflect the temporal realities of the systems they seek to understand. This may result in an over-reliance on short-term indicators or proxy measurements that may not adequately capture system performance, particularly in livestock-integrated systems where nutrient cycling and environmental outcomes evolve over extended periods. It may also limit the ability to detect unintended consequences or delayed responses associated with management interventions, particularly in relation to water quality and nutrient losses.

Addressing these challenges requires a shift in how agroecosystem research is conceptualized and supported. Rather than viewing long-term research as an extension of short-term studies, it is more appropriately understood as a form of research infrastructure, providing continuity, methodological consistency, and the capacity to integrate across spatial and temporal scales (Johnston and Poulton, 2018; Tsegaye et al., 2024). Such infrastructure must be supported through a combination of institutional commitment, networked collaboration, and funding models that recognize the value of sustained observation and cumulative knowledge generation. Platforms such as PFAR represent one response to this need. By embedding long-term measurement within production-representative farming systems and linking these measurements to broader research networks, PFAR enables the study of agroecosystem processes as they unfold in real-world contexts. In doing so, it provides a foundation for understanding not only how systems function in the short term, but how they evolve over time in response to interacting environmental, management, and socio-economic drivers.

## Platform Description and Research Infrastructure

The PFAR platform is located in Lafayette County, Wisconsin, USA (42.7125° N, 90.3987° W; 332 m a.m.s.l.), within the Upper Midwest region of the United States and the unglaciated Driftless Area (MLRA 105). This landscape is characterized by rolling topography, dense stream networks, and karst geology (Natural, 2022). These features create strong hydrological connectivity between fields, groundwater, and surface waters, making the region highly sensitive to land management decisions (Juckem et al., 2006).

Soils are predominantly Tama silt loams (TaC2 and TaB2 series), typical of upland Driftless Area systems. Topsoil (0–15 cm) pH ranges from 5.1 to 7.1, with organic matter content between 3% and 4% (Natural, 2026). On-site soil conservation practices include conservation tillage, contour strip cropping, grassed waterways, terracing, manure composting, and no-till planting. The site experiences a humid continental climate (Köppen–Geiger classification: Dfa), characterized by cold winters and warm, humid summers. Mean monthly temperatures range from −8 °C in January to 21 °C in July, with mean annual precipitation of ∼850 mm distributed throughout the year (NOAA, 2024).

The platform operates as a production-representative research farm embedded within this landscape. Rather than functioning as a controlled experimental station, PFAR mirrors regional cropping-livestock systems, allowing agronomic and livestock management decisions to be evaluated within environmental and operational context. Crops include corn, corn silage, oats, and alfalfa, for onsite livestock enterprises. These include a 200-cow Holstein and Brown Swiss dairy herd housed in a conventional free-stall system with robotic and double-5 herringbone milking parlors, a 50-head beef cow-calf herd, and a 70-head swine farrow-to-finish system. Livestock contribute to nutrient cycling primarily through feed production, manure collection, storage, and land application, linking animal production to soil fertility and environmental outcomes. Long-term experimental fields are managed under consistent, regionally relevant practices, enabling attribution of cumulative soil and hydrological responses to management while capturing interannual climate variability.

A defining feature of the platform is its integration of field-scale experimentation with continuous hydrological monitoring. With 21 active edge-of-field runoff stations, 26 vadose-zone lysimeters, and 11 groundwater wells providing direct measurement of nutrient and sediment fluxes across surface and subsurface pathways. Measured variables include nutrient concentrations, sediment loads, runoff volumes, flow dynamics, precipitation, and temperature. Soil health monitoring complements hydrological measurements through repeated assessment of physical, chemical, and biological indicators aligned with established soil health frameworks and harmonized with USDA LTAR protocols, including soil organic matter, bulk density, infiltration capacity, pH, and nutrient status. Together, these measurements support long-term agroecosystem assessment. This design enables evaluation of interactions among crop production, livestock management, nutrient cycling, and hydrological processes within landscape context, supporting both local relevance and broader networked synthesis.

## Hub-and-Spoke Network: Defining, Testing, and Scaling Sustainable Agriculture

**Figure 1.**
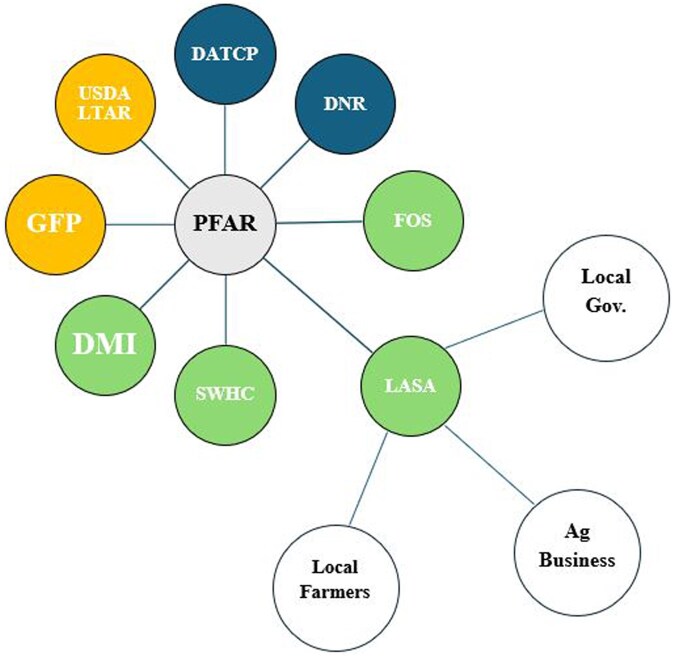
Conceptual illustration of the PFAR hub-and-spoke network as a transdisciplinary research and governance system. PFAR functions as a coordinating hub linking public agencies (e.g., Wisconsin Department of Agriculture, Trade and Consumer Protection [DATCP]; Wisconsin Department of Natural Resources [DNR]), research organizations (e.g., Global Farm Platform [GFP]; USDA Long-Term Agroecosystems Research [LTAR]), and farmer organizations (e.g., Dairy Management Incorporated [DMI]; Fields of Sinsinawa [FOS]; Lafayette Ag Stewardship Alliance [LASA]). The network operates through boundary-spanning roles and bridging ties that support knowledge co-production and bidirectional exchange between research, policy, and practice. Its fractal architecture repeats across scales, with organizations such as LASA functioning simultaneously as spokes and local hubs, enabling adaptive governance and scalable agroecosystem innovation.

The PFAR hub-and-spoke network integrates public agencies, research institutions, and farmer-led organizations, each contributing distinct but complementary capacities to agroecosystem research and implementation (Figure 1). Public agencies, including the Wisconsin Department of Agriculture, Trade and Consumer Protection and the Wisconsin Department of Natural Resources, utilize PFAR monitoring data to inform nutrient management regulations and decision-support tools.

At broader scales, PFAR functions as a contributing node within the USDA LTAR Network and the GFP. Alignment with LTAR core measurements and data standards enables cross-site comparison and national-scale synthesis, supporting assessments of climate resilience, nutrient cycling, and agroecosystem performance. Participation in the GFP extends this capacity to international benchmarking of agricultural systems.

Farmer-led watershed groups ground these networks in operational, social, and policy realities. Their involvement enhances both the relevance and credibility of research outputs, particularly for water quality, nutrient management, and ecosystem service outcomes that are governed at catchment scales (Richardson et al., 2022). These groups function simultaneously as local hubs, aggregating farmer participation, coordinating monitoring, and facilitating knowledge exchange, and as spokes within larger research networks, contributing data, experiential knowledge, and implementation capacity.

Within the PFAR platform, participating institutions contribute complementary roles that collectively support the research and implementation agenda. Research organizations provide experimental design, data integration, and analysis capacity, while public agencies contribute regulatory context, monitoring frameworks, and decision-support tools. Farmer-led watershed groups play a central role in coordinating participation, facilitating data collection, and grounding research activities within operational realities (Waters-Bayer et al., 2015). Interactions through working groups, advisory structures, and collaborative planning processes enable the co-development of research priorities and adaptive responses to emerging challenges. From these interactions, a set of implicit governance principles, or “design rules,” can be identified. The network operates through distributed leadership, shared data frameworks, aligned measurement, and iterative co-production of knowledge, enabling coordinated yet flexible agroecosystem research.

## Key Insights Emerging from Pioneer Farm Agroecosystem Research

A defining feature of PFAR is the direct linkage between management practices and measured environmental outcomes. Through continuous edge-of-field monitoring and associated hydrological measurements, PFAR enables assessment of how management decisions influence nutrient losses and water quality. This is particularly important in livestock-integrated systems, where nutrient flows are governed not only by crop uptake but also by the timing, placement, and redistribution of manure derived from animal production. In confined dairy systems, nutrient cycling is mediated through feed production, manure storage, and land application, rather than through grazing-based redistribution. Direct measurement supports transparent assessment of environmental performance, while revealing lagged responses and threshold behaviors in hydrologically connected landscapes.

Livestock-related nutrient dynamics further highlight the importance of cross-scale connectivity in agroecosystem research. Field-scale processes, including nutrient application and soil disturbance, influence catchment and regional-scale outcomes through hydrological pathways. In livestock-based systems, these linkages are intensified by spatial separation of feed production and manure application, which creates additional complexity in nutrient distribution (Garrett et al., 2020). The PFAR platform demonstrates how coordinated measurement frameworks connect field-scale processes with watershed-level outcomes and, through LTAR and GFP alignment, extend insights to national and international contexts. Social and institutional processes also shape agroecosystem outcomes. Farmer watershed groups ground research in operational realities while facilitating the translation of findings into practice. In livestock systems, constrained by infrastructure, labor, and regulation, co-production of knowledge is essential for ensuring management recommendations are scientifically robust and practically implementable. The temporal dimension of agroecosystem processes is another critical insight from the PFAR. Processes underpinning soil health, water quality, and system resilience develop gradually and are strongly influenced by interannual variability. Sustained measurement and institutional continuity are therefore necessary to detect legacy effects and understand responses to management and climatic variability.

Taken together, these insights position PFAR as a framework for agroecosystem research, designed to integrate across spatial and temporal scales and support the co-production of knowledge. Within this framework, livestock production is not treated as a discrete component but as an integral part of system function, influencing and being influenced by soil processes, hydrology, and management decisions. By embedding livestock within long-term research systems, PFAR improves understanding of how animal production influences both productivity and the environmental performance.

## Knowledge Exchange and Capacity: Data Management, Integration, and Accessibility

The platform employs standardized data collection protocols, quality control procedures, and metadata documentation to ensure consistency across datasets. Soil, water, management, and climate data are integrated for systems-level analyses with alignment to LTAR and GFP standards, facilitating cross-site synthesis and data sharing. Where possible, datasets are made accessible through network repositories, supporting transparency, reuse, and collaborative research.

Knowledge exchange is a core function of the PFAR, supporting translation of research into practice. Activities include producer field days, watershed reports, technical guidance for conservation agencies, and training opportunities for students and early-career researchers. Integration with agency tools and decision-support systems further enhances the uptake of research outputs, particularly in relation to nutrient management and water quality. For example, analysis of edge-of-field runoff data has shown that nutrient losses are highly sensitive to precipitation events occurring shortly after application, informing manure management practices and decision-support tools aimed at reducing nutrient loss risk. Key examples include the Wisconsin Phosphorus Index, the Wisconsin Run-Off Risk Advisory Forecast, the Annual Phosphorus Loss Estimator (APLE), and the Precision Agricultural-Landscape Modelling System (PALMS) (Table 1). Monitoring of feed production systems has also revealed spatial variability in nutrient losses linked to soil properties, landscape position, and management history, supporting targeted nutrient management and prioritization of conservation practices in high-risk areas.

**Table 1.** Role of Pioneer Farm Agroecosystem Research (PFAR) in advancing nutrient loss assessment tools and decision-support frameworks in agroecosystems

Framework/tool	Primary function	Core variables/processes	Role of PFAR (evidence and contribution)	Livestock system relevance	Limitation addressed by PFAR
Wisconsin Phosphorus Index (P Index)	Field-scale assessment of P loss risk to surface water	Soil test P, erosion, runoff potential, manure/fertilizer application timing and methods	Provide long-term, real field datasets to validate and refine P index coefficients under variable management and weather conditions	Captures manure P dynamic from dairy systems: links application timing to runoff events	Traditional P index often static and assumes average conditions; PFAR introduces temporal variability and event-based validation
Runoff Risk Advisory Forecast (RRAF)	Short-term (daily seasonal) forecasting of runoff risks to guide manure/fertilizer application decisions	Weather forecast, soil moisture, snowmelt, freeze-thaw cycles, rainfall intensity	Enables validation of forecast accuracy against edge-of-field runoff events; support calibration under real farm conditions	Critical for timing manure spreading in dairy systems to reduce nutrient losses	Forecast tools lack field validation; PFAR provides empirical datasets linking forecast to actual runoff outcomes
Annual Phosphorus Loss Estimator (APLE/APL)	Estimates long-term annual P loss from agricultural fields	Hydrology, erosion, dissolved and particulate P transport, management practices	Supports parameterization and testing of APLE under diverse rotation and management systems	Integrates manure P sources and livestock nutrient cycling over time	Often limited by simplified assumptions; PFAR adds multi-year; system-level variability and validation
Precision Agricultural Landscape Modelling System (PALMS)	High-resolution simulation of water, sediment, and nutrient transport across landscapes	Topography, soil properties, hydrological connectivity, management practices	Provides spatially explicit datasets to improve model realism and scaling from field to watershed	Represents livestock driven nutrient redistribution across landscapes (e.g., manure, feed production areas)	Models often lack calibration at multiple scales; PFAR integrates edge-of-field, farm, and watershed data
Edge-of Field Monitoring (PFAR Core Infrastructure)	Direct measurement of runoff volume and nutrient losses	Runoff flow, sediments total P, dissolved reactive P, nitrate-N, etc.	Generates high-frequency empirical data used to test, refine, and integrate all above tools	Directly quantifies nutrient losses from livestock-influenced systems	Addresses lack of real-world validation and long-term datasets in model and index development

Engagement with PFAR has enabled producers and scientists to field test alternative low-cost surface water monitoring options (Parker and Busch, 2013; Vadas et al., 2015) and interpret monitoring results in the context of working farms, leading to increased adoption of practices aimed at improving nutrient use efficiency and reducing environmental impacts. These activities demonstrate how the platform not only generates data but also supports learning, adaptation, and early identification of system responses to management.

## Limitations and Challenges: Value and Transferability

While the PFAR platform illustrates the value of hub-and-spoke agroecosystem research infrastructure, it also reveals governance, methodological, and institutional challenges associated with maintaining and scaling these systems. A central challenge concerns governance and power distribution within multi-institutional networks. Although hub-and-spoke systems require coordination, overly centralized decision-making can reduce responsiveness to the diversity of participating farms and stakeholders, particularly in livestock systems shaped by varying infrastructure, labor, and regulatory constraints.

Tensions also exist between methodological harmonization and site-specific flexibility. Participation in LTAR and GFP requires alignment with shared protocols and data standards to support cross-site synthesis. While essential for generating generalizable insights, this can constrain investigation of locally important processes, novel measurement approaches, and adaptive management responses. As a result, agroecosystem research must balance comparability with contextual relevance. Participation, equity, and representativeness present additional challenges. Farmer watershed groups operate under different institutional logics than academic or government organizations, and differences in timelines, data expectations, and definitions of success can create friction, particularly when monitoring requirements impose additional burdens on farm operations. Participating farms may also represent early adopters of conservation practices, raising questions regarding broader representativeness. Addressing these limitations requires strategies that broaden participation across more diverse production contexts.

The long-term viability introduces additional complexity. Hub-and-spoke systems depend on sustained coordination, data management, and stakeholder engagement, and institutional support, yet existing funding structures remain poorly aligned with the temporal requirements of agroecosystem research. This creates vulnerabilities related to leadership transitions, staffing continuity, and infrastructure maintenance, particularly where coordination capacity becomes concentrated within a limited number of individuals or institutions. To mitigate these risks, PFAR employs a polycentric governance structure in which authority and responsibility are distributed across research institutions, agencies, and farmer groups. This reduces reliance on a single coordinating entity while supporting broader participation, shared data stewardship, and co-development of research priorities. These approaches align with broader frameworks in transdisciplinary agroecosystem research that emphasize iterative leaning, integration of scientific and experiential knowledge, and adaptive governance. Taken together, these challenges do not diminish the value of hub-and-spoke systems, but highlight the importance of designing them with explicit attention to governance, flexibility, equity, and long-term resilience.

## Conclusions and Future Direction

The PFAR platform provides a foundation for long-term soil health, water quality, and farming systems research in Wisconsin, USA. By integrating long-term field experimentation, hydrological monitoring, stakeholder engagement, and networked collaboration, PFAR enables agroecosystem processes to be understood as interconnected systems. A central insight emerging from PFAR is that many processes underpinning soil health, nutrient dynamics, and environmental outcomes cannot be interpreted through short-term studies alone, but instead reflect cumulative interactions among management, climate variability, soil processes, and hydrological connectivity over extended timeframes.

The PFAR platform also demonstrates the importance of directly linking management and institutional context practices to environmental outcomes. Through continuous edge-of-field and hydrological monitoring, the platform provides a more transparent basis for evaluating nutrient losses and water quality within livestock-integrated systems. Integration of farmer watershed groups further grounds research within operational realities and supports co-production of management strategies that are both scientifically robust and practically adoptable.

More broadly, PFAR highlights the value of flexible, polycentric research systems capable of integrating scientific, institutional, and stakeholder knowledge across scales. Looking forward, continued development of PFAR and similar platforms will depend on sustained long-term measurement, advances in data integration and decision-support systems, and continued collaboration among researchers, agencies, and producers. As pressures associated with climate change, water quality degradation, and biodiversity loss intensify, integrated agroecosystem research infrastructures will become increasingly important for supporting resilient and environmentally sustainable agricultural systems.

## Author Biographies

**Dennis L. Busch** is a Senior Scientist at the University of Wisconsin-Platteville. His work centers on applied agroecosystem research, with emphasis on pasture- and crop-livestock systems, soil and nutrient dynamics, and long-term field experimentation. He plays a key role in developing and coordinating research platforms that integrate biophysical measurements with producer engagement, outreach, and stakeholder co-design. Dennis’s research bridges experimental science and on-farm decision-making, supporting practical innovations in agricultural hydrology, grazing management, soil health, and environmental stewardship. He is actively engaged in collaborative, multi-institutional efforts that translate research outcomes into extension and policy-relevant insights, strengthening the relevance of agroecosystem science for working landscapes and regional sustainability initiatives.

**Andrew D. Cartmill** is the Trevor Ellett Senior Lecturer in Grassland Science at Massey University, New Zealand. His research focuses on pasture-based livestock systems, integrating grassland ecology, forage quality, and soil-plant-animal interactions to enhance productivity, resilience, and environmental performance. He adopts systems-oriented and interdisciplinary approaches that link biophysical processes with farm management and policy-relevant outcomes, particularly in relation to climate mitigation, nutrient management, and ecosystem services. Andrew’s work contributes to national and international research networks and emphasizes the translation of environmental plant physiology and agroecology into decision-making frameworks for sustainable production from resilient agriculture systems. His research supports the development of future-ready livestock systems capable of responding to climatic, environmental, and socio-economic change.
